# Parental knowledge, attitudes, and practices toward antibiotic use for childhood Upper Respiratory Tract Infections in Khartoum, Sudan

**DOI:** 10.1186/s13756-025-01650-2

**Published:** 2025-10-28

**Authors:** Rowa A. Yousif, Ahmed K. Ali, Egbal A. Hassan, Tibyan N. Mohammed, Mohammed Almotasim S. Aldirdiri, Elfatih M. Malik

**Affiliations:** Faculty of Medicine, University of Khartoum, Khartoum, Sudan

**Keywords:** Knowledge, Attitude, Perception, Practice, Antibiotic use, Antimicrobial resistance, Sudan

## Abstract

**Background:**

Upper respiratory tract infections (URTIs) are among the most common diagnoses resulting in antibiotic prescriptions. Reducing unnecessary antibiotic use is critical to avoiding irrational use and antimicrobial resistance; one of the top 10 global public health concerns facing humanity. As monitoring and administering therapy for children’s ailments are mostly the responsibility of their parents, their perception will have a significant effect on whether or not it is administered appropriately.

**Methods:**

This cross-sectional hospital-based study was conducted at 2 major children teaching hospitals (Ibrahim Malik and Jaafar Ibn Auf) in Khartoum, Sudan. A simple random sampling was applied and a questionnaire adapted from a previous study was used to collect the data. Data was analyzed using a statistical package for social sciences (SPSS). Ethical clearance was obtained from the Department of Community Medicine, Faculty of Medicine, University of Khartoum, the ministry of health, and the hospitals in which the study was conducted.

**Results:**

Two hundred and eleven out of two hundred thirty-nine randomly selected parents participated in the study (response rate of 88.3%). Although, 63.5% of parents were aware that antibiotics should not be administered for every case of fever, only 39.3% of respondents were aware that URTIs are caused by viruses and do not require antibiotic therapy. 60% (60%) believed that antibiotics cure URTI symptoms faster and 70.6% were unaware that inappropriate antibiotic administration leads to bacterial resistance. The most prevalent symptom that prompts parents to seek medical assistance was earache (80.6%), followed by fever and sore throat (68.2% and 64.5%, respectively). When they went to the doctor, 73.3% expected antibiotics to be prescribed, and more than 65% wanted antibiotics to be administered if their child had cold or nasal drainage. Only 28.4% of parents said they never administer antibiotics without consulting a doctor.

**Conclusions:**

Parents should be educated about the duration of URTIs, the self-limiting nature of such infections in children, and how to use antibiotics safely and effectively. The provision of such knowledge may minimize parents’ fears and concerns regarding URTIs, hence reducing antibiotic use.

## Introduction

Sudan is the third-largest country in Africa, with a population of over 40 million. Of those, more than half are children [1], making the health of Sudanese children a critical concern and addressing common health issues like upper respiratory tract infections (URTIs) becomes even more crucial. Among URTIs rhinitis is the most prevalent type [2]. These infections are primarily caused by viruses, with rhinoviruses being the most common [3].

In parallel, antimicrobial resistance (AMR) is a significant and growing threat in Sudan’s healthcare system, with a high prevalence of multidrug-resistant bacteria [4]. AMR is exacerbated by factors such as self-medication and poor adherence to treatment guidelines [5]. The financial burden of AMR is also substantial, with high healthcare costs and increased hospital admissions [6]. The global nature of this problem further underscores its severity [7].

In pediatrics, the practice of prescribing antibiotics for URTIs is widespread, despite the fact that the majority of these infections are viral in nature. Antibiotic resistance is largely caused by the over prescription of antibiotics, an issue exacerbated by both parents and pediatricians [8]. The misuse or abuse of antibiotics has brought up a number of concerns, including rising healthcare costs, an increase in side effects like diarrhea, and a rise in antibacterial resistance. Antibiotic misuse and the emergence of resistance are closely linked, and nations with high antibiotic usage rates have also been found to have higher incidences of resistant infections [9].

The role of parents in the use of antibiotics is considerable. The majority of parents had inadequate information, a poor attitude, and poor practices regarding their children’s use of antibiotics. The appropriate use of antibiotics in children attending clinics in Tanzania was influenced by the parents’ work position, education level, and good attitude [10]. Cultural aspects, and behavioral traits including self-medication, educational attainment, and financial standing are all associated with antibiotic misuse. A significant contributing factor to the abuse of antibiotics is also a lack of health education [11].

To reduce resistant bacteria, a number of strategies must be employed, such as the judicious use of antibiotics. These strategies need to take a comprehensive approach, considering important stakeholders such as scientists, policymakers, donors/funders, and implementers like the community, parents/guardians, and healthcare providers. Several measures are being used at the implementation level, such as AMR stewardship programs that encourage the careful use of antibiotics, monitoring and evaluation of antibiotic usage in healthcare facilities, training healthcare personnel, raising community awareness, and the One Health initiative [12]. The experience of the kingdom of Saudi Arabia underscored the necessity of stricter laws governing doctors’ prescribing practices for antibiotics as well as parental education initiatives on antibiotic use [13].

There is limited information on parental knowledge, attitudes, and practices toward antibiotic use for URTIs among children in Sudan. Understanding these factors is essential to guide educational and policy interventions. Therefore, this study aimed to assess the knowledge, attitudes, and practices of parents regarding antibiotic use for upper respiratory tract infections in children in Khartoum, Sudan.

## Materials and methods

### Study design and settings

This observational cross-sectional study was conducted at Ibrahim Malik Teaching Hospital and Jaafar Ibn Ouf Children`s Hospital, the largest and popular hospitals in Khartoum state with a pediatrics department. Ibrahim Malik Hospital offers different medical specialties and services for more than 100,000 population, it contains 299 patient beds, is affiliated with the International University of Africa and the University of Khartoum, recognized by the Sudan Board for Medical Specialization and the Federal Ministry of Health for the training of registrars and house officers.

Khartoum Children’s Reference Hospital (Jaafar Ibn Auf) is located on Almak Nimr Avenue, Khartoum. It includes 193 beds, 25 nurseries, and intensive care for newborns, a hemodialysis unit, gastrointestinal unit, hematology unit, endocrine unit, neurology unit, respiratory unit, cardiology, and an ultrasound unit. It accepts referred cases from specialists in other hospitals and the referring clinic.

## Study population and sampling

The study population were parents who attended Ibrahim Malik Teaching Hospital and Jaafar Ibn Auf Children`s Hospital in the period between the 28th of February to the 10th of March 2022. Any parent who has a child under 18 years old was included and the only exclusion criteria was to be a non-Sudanese attendee.

The sample size was calculated to be 237 using the formula (n = Z^2^pq/e^2^) where:

n = minimum sample size required, z = probability that e is not exceeded (95% confidence level), p = expected prevalence of URTIs (found to be 19 from Sudan’s annual health statistical report 2020), q = 1-p, e = maximum acceptable random sampling error (here is 5%).

Sampling was made using a simple random sampling method.

## Data collection

The data was collected using a questionnaire adapted from a previous study on the same topic conducted in Greece [14], it was translated into Arabic and reviewed by a specialist. The translated questionnaire was converted into a Google form filled out by interviewing the participants by the researcher The independent variables include: age, gender, educational level, economic status, and residency. The dependent variables are the knowledge, attitude, and practice of parents attending the two hospitals toward antibiotic use for upper respiratory tract infections.

## Data management and analysis

The data entry and analysis were processed using Statistical Package for Social Science (SPSS) ® Version No. 24 software for statistical analysis.

The knowledge score was calculated to be as high as 35 for the participants who answered all the 7 questions correctly, 7 was the least score, and 21 was set as the cut-of-point between good and poor knowledge. The attitude and practice scores were calculated to be as high as 30 for the best answers for all the 6 questions, 6 was the least score, and 18 was set as the cut-of-point between good and poor attitude and practice.

Simple descriptive statistics: Frequencies, percentages, and chi-square tests were used. The results were displayed in tables and graphs.

Ethical considerations: Ethical approval was obtained from the Department of Community Medicine, Faculty of Medicine, University of Khartoum. A permission to conduct the study was taken from the Ministry of Health and the hospitals directors in which the study was conducted. Also, written voluntary informed consent was obtained from the participants before interview.

## Results

Two hundred and eleven out of two hundred thirty-nine randomly selected parents agreed to participate in the study giving a response rate of 88.3%. (*n* = 239)

### Socio-demographic characteristics of the study population

The majority of respondents (77.3%, *n* = 211) were females, with a mean (± SD) age of 36 ± 9.8 years. Most of them (87.7%) were married at the time of data collection. Approximately two-thirds (64.4%) of them lived in the town. Nearly (93%) of the participants didn’t work in the medical field, and only (21.8%) had a child suffering from a chronic Respiratory condition. The income, Father’s and mother’s educational status are shown in Table 1.

**Table 1:** Socio-demographic characteristics of the participants (*n* = 211)

Variable	Variable description	Frequency	Percentage
Sex	Male	48	22.7
	Female	163	77.3
Marital status	Married	185	87.7
	Divorced	16	7.6
	Widow	10	4.7
Residence	Town	137	64.9
	Village	74	35.1
Father’s educational status	Uneducated	30	14.2
	Basic school	105	49.8
	University and above	76	36.0
Mother’s educational status	Uneducated	37	17.5
	Basic school	103	48.8
	University and above	71	33.7
Health insurance	Public	53	25.1
	Private	14	6.6
	No insurance	144	68.2
Family’s monthly income	Low income (< 50000 SDG)	78	37.0
	Moderate income (50000–100000 SDG)	86	40.8
	High income (> 100000 SDG)	47	22.3
Parent work in medical field	Yes	15	7.1
	No	196	92.9
Have child with a chronic Respiratory condition	Yes	46	21.8
	No	165	78.2

## Knowledge

Regarding the primary source of knowledge about true antibiotic use, most of the parents 74.4% stated that their physician told them and the most antibiotic in use is amoxicillin /clavulanic acid (Table 2).

**Table 2:** Sources of information about judicious antibiotic use and parents recognition of antibiotics

Variables	Description of variables	Frequency	%
Sources of information about judicious antibiotic use	Physician	157	74.4
	Pharmacist	96	45.5
	Television and Radio	14	6.6
	Newspapers	5	2.4
	Friends and Family relatives	20	9.5
	Internet	2	0.9
Number of parents that recognize this is an antibiotic	Ibuprofen	51	24.2
	amoxicillin /clavulanic acid	165	78.2
	Paracetamol/pseudoephedrine hydrochloride/diphenhydramine hydrochloride	67	31.8
	Cephalexin	69	32.7
	Diphenhydramine HCl/sodium citrate/ menthol	69	32.7
	Azithromycin	133	63.0
	Couldn’t recognize any	17	8.1

## Discrimination between antibiotic products and other drugs

When parents were asked to discriminate between antibiotic products and other drugs, including analgesics and antipyretics and cough preparations and expectorants most parents (78.2%and 63.0%) were able to identify that amoxicillin/clavulanic acid and Azithromycin were antibiotics, while (32.7%) were able to identify that Cephalexin is an antibiotic. Sadly, about one thirds, (32.7% and 31.8%) of parents identified cough preparation combinations diphenhydramine HCl/sodium citrate/menthol and paracetamol/pseudoephedrine hydrochloride/diphenhydramine hydrochloride as antibiotics, respectively. However, (8.1%) were not able to recognize any of the drugs as an antibiotic (Table 2).

### General knowledge

A total of (63.5%) of parents were against Antibiotic administration for every case of fever, however, only (39.3%) were attentive to the truth that the majority of URTIs are of viral cause and necessitate no antibiotic treatment, and approximately (60%) thought that antibiotics cure URTIs symptoms quicker. (70.6%) agreed with the fact that improper administration of antibiotics decreases their efficacy and causes bacterial resistance. Only (25.1%) agreed that Antibiotics do not present side effects and (58.3%) were aware that they might cause side effects, moreover, (62.5%) believed that Antibiotics decrease the complications of an URTI, and (59.3%) decided that Scientists can always produce new antibiotics that are able to kill the resistant bacteria.

Knowledge score:

Only 63 (29.9%) of the participants have good knowledge about the use of antibiotics for URTIs and the socio-demographics had no association with the knowledge score.

### Attitude

Days parents let pass before seeking a doctor’s advice:

Parents were asked how many days would you let pass to visit a pediatrician, if their child presents some symptoms (i.e. Nose drainage, sore throat, vomit, cough, fever)? And more than 87% would seek a doctor’s advice in less than 3 days (mean duration 2.3 days; SD = 1.5).

Parent’s expectations about the medication for their child’s URTIs.

When parents were provided with possible treatment options for the managing URTIs, (73.0%) of them chose antibiotics therapy, and (45.0%) of parents choose analgesics and antipyretics as a possible treatment option for URTIs, while (34.6%) of the participants choose Antitussives as a possible therapy. Only 12.3% choosed antihistamines as a possible therapy.

**Fig. 1:**
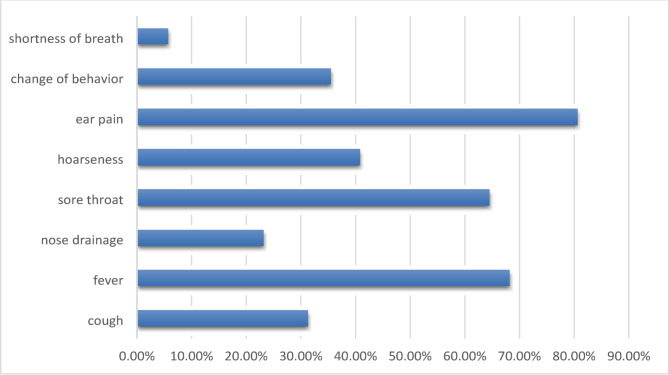
symptoms that would make the parent visit a pediatrician for their child

Symptoms that cause parents to visit a doctor:

Earache was the most common symptom (80.6%) which causes parents to seek physician’s advice, followed by fever and sore throat (68.2% and 64.5%), respectively (Fig. 1).

Questions related to attitude:

The majority of parents (71.7%) agreed that antibiotics are extensively used. nearly (60%) of the participants stated that they wouldn’t change their pediatricians if they did not prescribe antibiotics frequently enough, yet, (51.7%) noted that they would switch pediatricians if antibiotics were prescribed so often, and (69.2%) indicated that they would not reuse leftover antibiotics that were prescribed for the same symptoms before. Nevertheless, nearly half of the participants thought that URTIs can’t be cured by themselves without the use of antibiotics. (77.3%) Thought that additional information should be provided for parents and pediatricians concerning adequate antibiotic use.

### Attitude score

Only 67 (31.8%) have good attitude about the use of antibiotics for URTIs.

There is a significant relation between being a female and having poor attitude **(p = .042)**. Other Socio-demographics’ have no relation with the attitude score.

### Practice

Administration of antibiotics without medical advice.

The most common cause for over-the-counter use of antibiotics was pharmacist recommendations (56.9%), followed by former prescriptions by a physician for the same symptoms and the belief that their child’s condition was not serious enough (43.1% and 38.4%), respectively (Table 3).

**Table 3:** Giving your child antibiotics without a pediatrician’s advice can be harmful and unnecessary

		*N*	Percent
Why would you give your child antibiotics without the pediatrician’s advice?	Because you did not have enough spare time to visit a pediatrician, or because you did not have enough money to pay the visit.	63	29.9%
	Because you thought that your child’s condition was not serious enough	81	38.4%
	Because your pediatrician had prescribed the same antibiotic in the past, for the same symptoms.	91	43.1%
	Because a pharmacist recommended the antibiotic.	120	56.9%
	Because a friend/ family relative recommended the antibiotic	21	10.0%
	I never give antibiotics without advice	60	28.4%

### Questions related to practice

About (40%) declared that they always ask their pediatrician wither antibiotics were essential, but (33.6%) never did. More than half of the parents stated that they would praise pediatricians if they choose not to prescribe antibiotics, while (31.8%) were more often to ask for an antibiotics prescription frankly. However, (85.3%) confirmed that they follow pediatrician’s tips precisely. About (23%) said that doctors explain to them if their child should or should not take antibiotics less than often, and (22.7%) of parents thought that their pediatricians prescribed antibiotics based on their request.

### Practice score

About half of them 115 (54.5%) have good practice about the use of antibiotics for URTIs. Socio-demographics’ data were not associated with the practice score, except for the parents aged less than 35 years and having good practice **(p = .000) **.

## Discussion

The observational cross-sectional study aimed to describe parental knowledge, attitudes, and practices towards the use of antibiotics for URTIs. It was conducted in two major hospitals in Khartoum, Sudan. A total of 211 attending parents agreed to participate in the study, with the majority being females (77.3%, *n* = 211) as mothers commonly accompany their children during hospital visits.

According to the literature [14–19], when participants were asked about their most reliable source of information regarding wise antibiotic use, nearly three-quarters chose their physician as the main source, followed by their pharmacist (45.4%). These findings indicate that the trust placed in these health professionals is crucial in educating and reassuring parents when faced with URTI symptoms. Additionally, more than half of the parents considered pediatricians who choose not to prescribe antibiotics praiseworthy, while one-third accepted that their pediatricians don’t prescribe antibiotics often enough. Surprisingly, over 30% of parents never ask their pediatricians whether antibiotics are necessary or not. However, half of them stated that they would change doctors if prescriptions were too frequent. These findings differ from those found in Palestine [16], where only 27% would change pediatricians due to perceived over prescription of antibiotics, and over two-thirds wouldn’t consider changing doctors if they believed that antibiotics aren’t prescribed enough. Sudanese parents tend to become anxious quickly about their child’s health when URTI symptoms appear, with over 87% seeking a doctor’s advice within three days of symptom onset. Parents in Saudi Arabia [19] and Singapore [20] exhibit similar behavior. Earache was the most common symptom that caused parents to seek a physician’s advice, followed by fever and sore throat. In Saudi Arabia [18], fever; in Greece [14], runny nose; and in Singapore [20], cough were the primary reasons for parents to seek medical attention.

Over half of the parents believed that antibiotics reduce URTI complications, consistent with findings in Singapore [20] and Palestine. However, this variation may be attributed to cultural differences among the survey participants. 60% of the parents confirmed that doctors explain the condition and treatment options, similar to findings in China [17] and Greece [14], but 50% in Saudi Arabia stated that doctors never provide such explanations. Consequently, nearly 80% of parents mostly follow the doctor’s advice, similar to Greece [14] and Palestine, but far from Saudi Arabia [18], where only 1% do so. More than 65% of parents wanted antibiotics to be prescribed if their child had a cold or nasal drainage. However, 31.8% explicitly requested antibiotics, and 22.7% thought that their pediatricians prescribed antibiotics based on their requests, similar to findings in Malaysia [21] and Palestine [16]. This is concerning as it suggests pressure on physicians to prescribe antibiotics even when they are not indicated. Over three-quarters of parents correctly identified amoxicillin/clavulanic acid, Azithromycin, and cephalexin as antibiotics, consistent with findings in China [22] but higher than those in Saudi Arabia [18] and Palestine [16]. A significant percentage of parents also considered cough preparations and antipyretics to be antibiotics, indicating confusion.

Regarding knowledge, approximately two-thirds of parents were against antibiotic administration for every case of fever, similar to Greek [14] and Saudi parents [18, 19] opinions but different from Palestinian parents [16] opinion, where 59.4% believed the contrary. However, almost 40% of parents were unaware that URTIs are of viral cause and do not necessitate antibiotic treatment. Moreover, over half of the parents wrongly believed that antibiotics cure URTI symptoms more quickly.

This misconception is shared with parents in China [17, 23] and, to a greater extent, with parents in Singapore [20] and Lebanon [24]. However, it was noticeably less prevalent in Palestine [16].

More than half of the participants recognized the issue of antibiotic side effects, similar to findings in China [22], Kosovo [15], Palestine [16], and Singapore [20]. Yet, more than two-thirds disagreed with the fact that improper antibiotic administration reduces their efficacy and causes bacterial resistance. This differs from findings in Palestine [16], Singapore [20], Greece [14], and Lebanon [24], but aligns with Kosovo and Malaysia. Furthermore, 59.3% of the participants believed that scientists can always produce new antibiotics capable of killing resistant bacteria, which is significantly less than what was found in China [17] and Palestine [16] but higher than in Saudi Arabia [18].

More than two-thirds of parents thought that antibiotics are overused, similar to findings in Greece [14] and Palestine [16]. Nonetheless, when presented with possible treatment options for URTI management, about three-quarters of parents expected to be provided with antibiotics, similar to the findings of Panagakou et al. Surprisingly, over 40% of parents did not want antibiotics prescribed for their children when they had ear pain. However, there was a relatively high desire for antibiotics if the child experiences nasal drainage, vomiting, a cold, or fever. This suggests the possibility of over-prescription rather than parental preference as the cause of antibiotic overuse. It is worth mentioning that, similar to the Greece study, over three-quarters of participants wanted additional information provided to parents and pediatricians concerning appropriate antibiotic use. However, like the Singapore [20] study, nearly half of the participants did not believe that URTIs can be cured without the use of antibiotics. Additionally, over 70% of parents admitted to administering antibiotics to their child without a prescription, compared to 40% in China [17, 22], the United Arab Emirates [25], and Saudi Arabia [18], and only 10% in Greece and 20% in Malaysia. Furthermore, a quarter of participants indicated that they would reuse leftover antibiotics previously prescribed for the same symptoms. This aligns with findings in China [26] and Malaysia [21] but exceeds the results of Singapore [20]. Over-the-counter use was typically guided by pharmacist recommendations, a previous prescription for similar symptoms, or the perception that the child’s condition was not serious enough, similar to findings in Saudi Arabia [18, 19]. However, only 10% resorted to over-the-counter use based on advice from friends or relatives.

## Conclusion

The physicians and pharmacists are trusted sources of information regarding the use of antibiotics in treating URTIs, and Sudanese parents do follow their instructions in this regard.

Most Sudanese parents visit the physician within three days of the onset of symptoms, and over three-quarters of them assume that antibiotics will be prescribed.

A large proportion of parents are not aware of the emergence of antibiotic resistance due to improper use, and 70% self-medicate their child with antibiotics.

Over-the-counter use is driven by pharmacist advice, a previous prescription for similar symptoms, and the belief that the child’s illness is not serious enough.

### Recommendations

Further research is needed to assess parents’ expectations and to determine the most effective strategies for educating them on the proper use of antibiotics in the treatment of URTIs. Additionally, further study is needed to better understand physicians’ attitudes and practices regarding antibiotic prescribing.

Parents should be educated about the duration of Upper respiratory tract infections, the self-limiting nature of such infections in children, and how to use antibiotics safely and effectively. Providing this knowledge may help alleviate parents’ fears and concerns about URTIs, thereby reducing antibiotic use.

We also recommend real-time doctor-specific prescription monitoring, and the development of algorithms to guide clinical decision-making.

## Data Availability

Data supporting the findings of this study are available and can be provided upon reasonable request.

## Data availability

Data supporting the findings of this study are available and can be provided upon reasonable request.
